# The short-term association of selected components of fine particulate matter and mortality in the Denver Aerosol Sources and Health (DASH) study

**DOI:** 10.1186/s12940-015-0037-4

**Published:** 2015-06-06

**Authors:** Sun-Young Kim, Steven J. Dutton, Lianne Sheppard, Michael P. Hannigan, Shelly L. Miller, Jana B. Milford, Jennifer L. Peel, Sverre Vedal

**Affiliations:** Department of Environmental and Occupational Health Sciences, University of Washington School of Public Health, Seattle, WA USA; Institute of Health and Environment, Seoul National University, Seoul, Korea; National Center for Environmental Assessment, U.S. Environmental Protection Agency, RTP, NC, USA; Department of Biostatistics, University of Washington School of Public Health, Seattle, WA USA; Departments of Mechanical Engineering, College of Engineering and Applied Science, University of Colorado, Boulder, CO USA; Department of Environmental and Radiological Health Sciences, Colorado State University, Fort Collins, CO USA

**Keywords:** Chemical components, Fine particulate matter, Mortality, Time-series study

## Abstract

**Background:**

Associations of short-term exposure to fine particulate matter (PM_2.5_) with daily mortality may be due to specific PM_2.5_ chemical components. Daily concentrations of PM_2.5_ components were measured over five years in Denver to investigate whether specific PM_2.5_ components are associated with daily mortality.

**Methods:**

Daily counts of total and cause-specific deaths were obtained for the 5-county Denver metropolitan region from 2003 through 2007. Daily 24-hour concentrations of PM_2.5_, elemental carbon (EC), organic carbon (OC), sulfate and nitrate were measured at a central residential monitoring site. Using generalized additive models, we estimated relative risks (RRs) of daily death counts for daily PM_2.5_ and four PM_2.5_ component concentrations at single and distributed lags between the current and three previous days, while controlling for longer-term time trend and meteorology.

**Results:**

RR of total non-accidental mortality for an inter-quartile increase of 4.55 μg/m^3^ in PM_2.5_ distributed over 4 days was 1.012 (95 % confidence interval: 0.999, 1.025); RRs for EC and OC were larger (1.024 [1.005, 1.043] and 1.020 [1.000, 1.040] for 0.33 and 1.67 μg/m^3^ increases, respectively) than those for sulfate and nitrate. We generally did not observe associations with cardiovascular and respiratory mortality except for associations with ischemic heart disease mortality at lags 3 and 0–3 depending on the component. In addition, there were associations with cancer mortality, particularly for EC and OC, possibly reflecting advanced deaths of a frail population.

**Conclusions:**

PM_2.5_ components possibly from combustion-related sources are more strongly associated with daily mortality than are secondary inorganic aerosols.

**Electronic supplementary material:**

The online version of this article (doi:10.1186/s12940-015-0037-4) contains supplementary material, which is available to authorized users.

## Background

Recent observational epidemiologic studies have focused on the adverse health effects of outdoor fine particulate matter (PM_2.5_) chemical components in order to identify the most toxic components responsible for the association between PM_2.5_ and human health. Most of these studies investigated the short-term association of exposures to PM_2.5_ components on a given day and counts of deaths or hospital admissions on the current or a few following day. Increases in daily concentrations of combustion-related components such as elemental and organic carbon were associated with increased cardiovascular mortality within a few days in California, New York City, and Phoenix [1–3], and total non-accidental and cardiovascular mortality in the cold season in Seattle [4]. Secondary aerosols such as sulfate and nitrate were also associated with total or cardiovascular mortality in California [1].

These studies of short-term associations for PM_2.5_ components have almost always made use of regulatory monitoring networks for the data on PM_2.5_ component concentrations [1, 2, 5–7]. While data from these networks are readily available and have proven to be useful for epidemiologic studies, such data are hampered by limited availability, frequency, and locations of measurements as well as the species being measured. For example, most regulatory monitoring networks have sampled PM_2.5_ components on a regularly missing schedule such as every 3^rd^ or 6^th^ day [8, 9]. These limitations may affect health effect analyses that aim to gain insight into features of PM_2.5_ that are more toxic [10, 11].

The Denver Aerosol Sources and Health (DASH) study was designed to enable investigation of the health effects of short-term exposure to chemical components of PM_2.5_ measured daily for an extended time period at a representative monitoring site for population exposure [12]. Here we report findings from the DASH study on associations of daily concentrations of PM_2.5_ and four PM_2.5_ components (EC, OC, sulfate and nitrate) with daily mortality attributed to the most common causes of death in the 5-county Denver metropolitan area over the 5 years of the study.

## Methods

### PM_2.5_ collection and characterization

**Fig. 1:**
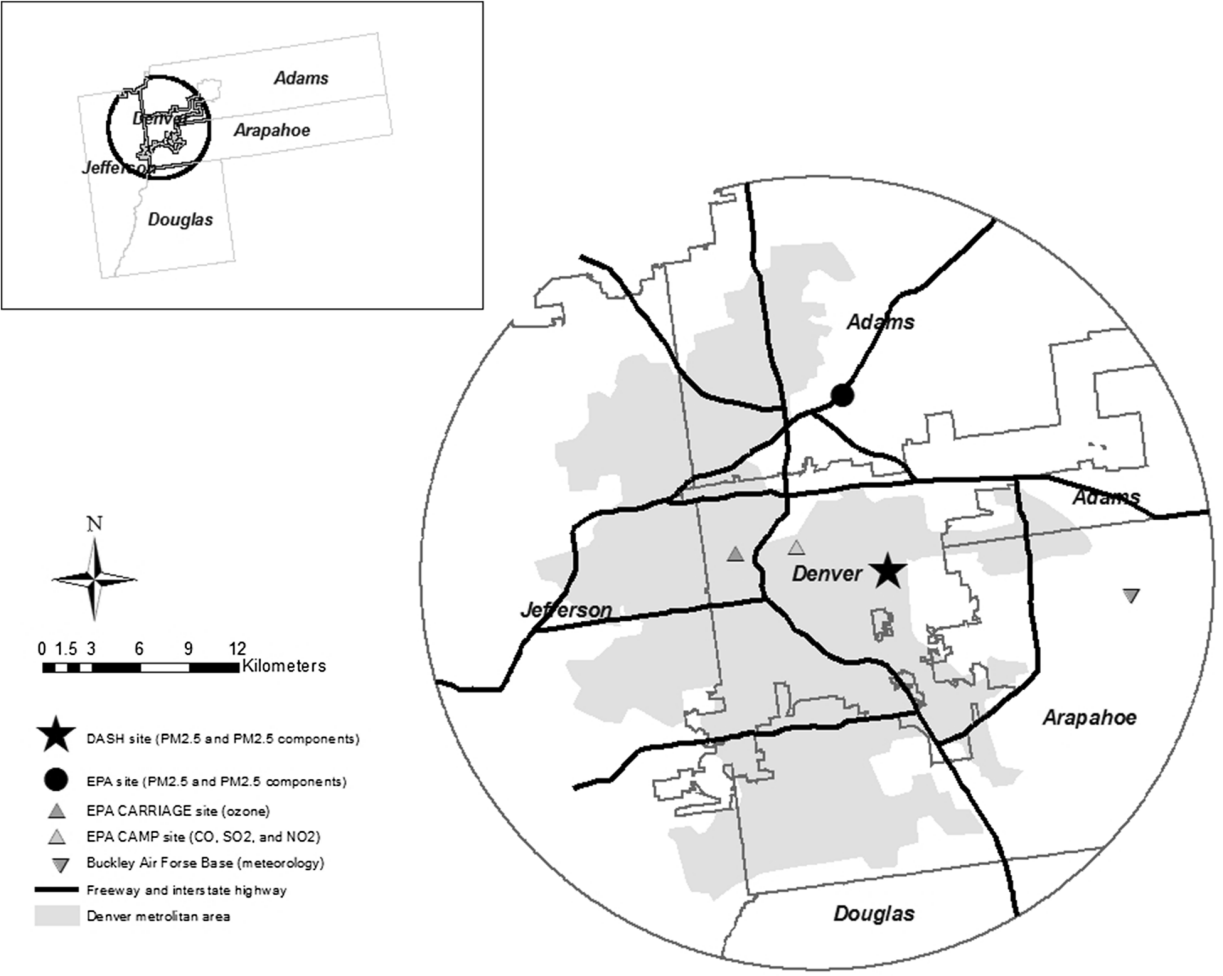
Map of the central area of the 5-county Denver metropolitan region showing major freeways and the location (⋆) of the residential monitoring site in the DASH study (● is the location of the EPA Chemical Speciation Network site)

The monitoring site, sampling equipment, and procedures, including quality assurance and quality control procedures, have been previously described in detail [12, 13]. Briefly, a residential monitoring site on the roof of an elementary school located 5.3 kilometer east of downtown Denver was constructed for obtaining the detailed PM_2.5_ chemical speciation data (Fig. 1). The site was selected for its central location in a large residential community and for its distance from point and mobile sources. The school is 5.2 kilometer from the closest large highway and at least 0.6 kilometer from the closest commuter street. Collection of daily 24-hour composite PM_2.5_ samples from this site was carried out from 2003 through 2007.

PM_2.5_ samples were collected daily from midnight to midnight with samplers operating at a flow rate of 92 L/min. PM_2.5_ was collected and sized by a sharp cut cyclone on two filter types: a Teflon filter used for gravimetric and inorganic analyses and a quartz fiber filter for carbonaceous component analyses. The volume of air sampled by each filter was measured using a calibrated dry gas meter. Bulk chemical speciation of all samples included the quantification of PM_2.5_ mass, inorganic ionic compounds (sulfate and nitrate), elemental and organic carbon (EC and OC). Gravitational mass measurements were made using a microbalance housed in a temperature and humidity controlled chamber. Filters were collected from the field within 72 hours of sampling and post-weighed within 2 weeks of collection. Inorganic ionic compounds were quantified using an ion chromatograph following standard methodology for airborne particulate matter. EC and OC concentrations were determined by the National Institute for Occupational Safety and Health Method 5040.

### Meteorology and gaseous pollutants

Meteorological measurements were collected at the Buckley Air Force Base about 14 kilometer away from the DASH monitoring site. We computed daily averages of temperature, relative humidity, and barometric pressure. Hourly measurements of gaseous pollutants at the two U.S. Environmental Protection Agency (EPA) monitoring stations in Denver were extracted from the U.S. EPA Air Quality System database. CO, SO_2_, and NO_2_ were collected at one EPA site 5.7 kilometer away from the DASH site, whereas O_3_ was measured at another site 9.2 kilometer away. Daily maximum of 1-hour averages for CO, SO_2_, and NO_2_, and daily maximum of 8-hour averages for O_3_ were computed.

### Health endpoints

Human subjects study approval was obtained from the Human Subjects Division, University of Washington. Daily mortality counts for metropolitan Denver were computed from the Colorado Health Information Dataset compiled by the Colorado Department of Public Health and Environment. Data included underlying cause of death by the International Classification of Diseases 10th Revision (ICD-10) code, residence zip code, gender and age at death. Deaths were restricted to the five-county Denver metropolitan region that includes Adams, Arapahoe, Denver, Douglas and Jefferson counties (Fig. 1). Total deaths were counted excluding accidental deaths with the ICD-10 codes beginning with ‘V’, ‘W’, ‘X’, ‘Y’, and ‘Z’. The three most frequent cause-specific deaths were defined based on the ICD-10 codes beginning with ‘I’, ‘J’, and ‘C00-D48’, for cardiovascular, respiratory and cancer deaths, respectively. We also included ischemic heart disease (IHD) mortality (ICD-10 code ‘I20-I25’) as a cause-specific mortality since previous time-series studies have found IHD to have the strongest associations with PM_2.5_ [14].

### Statistical analysis

The associations between the daily PM_2.5_ mass and chemical component concentrations measured over the 5-year period and the daily mortality counts were assessed using generalized additive models [15]. In these models, we adjusted for longer-term temporal trend, as time since the study began, day of week, and daily temperature and humidity. Non-linear trend of the associations of time and temperature with mortality was accounted for by regression splines with 60 (12 per year) and 3 degrees of freedom (df), respectively. In order to capture the pollutant effect of different days, we estimated effects of pollutant concentrations for 0 to 3 days (lags 0, 1, 2, and 3) preceding the day of death. In addition, the effect of pollutant concentrations distributed over several days from lag 0 through 3 (lag 0–3) was estimated from an unconstrained distributed lag model [16]. Estimated effects of PM_2.5_ and PM_2.5_ components on mortality were presented as relative risks (RRs) and 95 % confidence intervals (CIs) for inter-quartile (IQR) increases in pollutant concentrations.

Several sensitivity analyses were carried out to assess the robustness of the findings to modeling choices. We examined smaller or larger df (20, 30, 40, 80, and 120) for long-term temporal trend, matching lags of meteorology to those of pollutants, and a squared term and moving averages of extended days (lags 0, 1–3, and 4–7) for temperature. We additionally adjusted for barometric pressure, the other PM_2.5_ components, PM_2.5_, and gaseous pollutants including CO, SO_2_, NO_2_, and O_3_. For PM_2.5_, we used residuals from the regression of PM_2.5_ on each PM_2.5_ component [17]. To assess whether the estimated effects were modified, analyses were repeated within age (<65 and ≥ 65), sex, and season (summer, winter, and spring-fall) strata. We also restricted our study area to 10 and 20 kilometer buffers from the monitoring site. Finally, in order to investigate the lag structure, we estimated effects at lag 0 through 14 in constrained distributed lag models [18, 19]. The constrained distributed lag model was specified based on B-splines with 5 df generally providing the lowest Akaike Information Criterion.

## Results

**Fig. 2:**
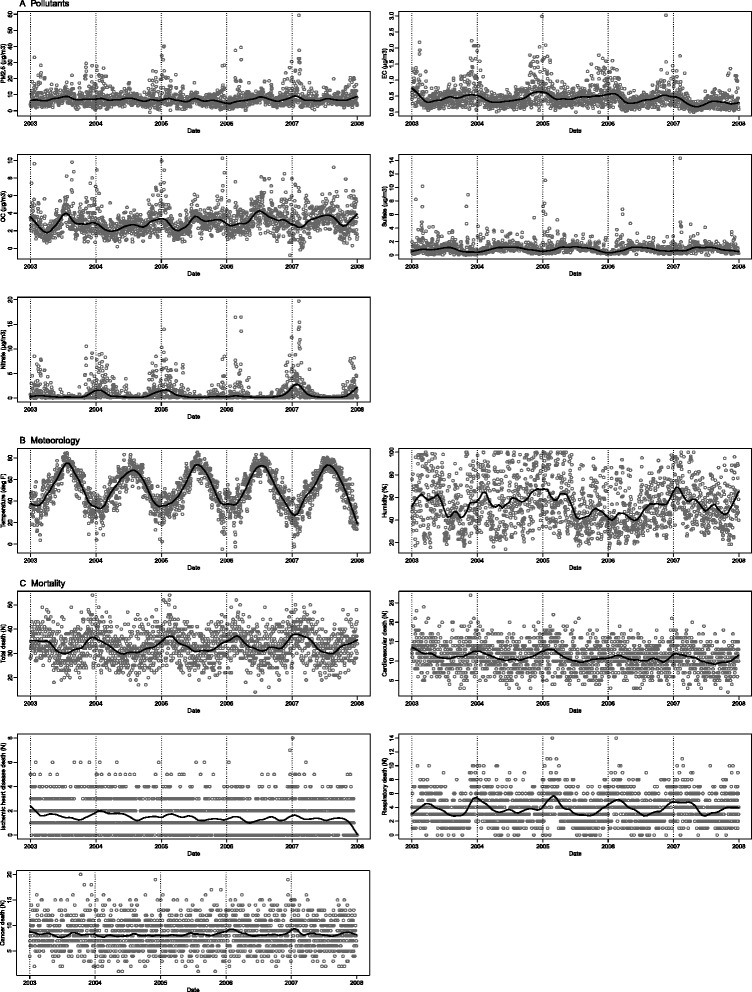
Time-series plots of (A) daily PM_2.5_ and four PM_2.5_ component concentrations, (B) meteorology, and (C) mortality in the 5-county Denver metropolitan region from 2003 through 2007 (smoothed lines based on locally-weighted polynomial regression)

Distributional statistics for PM_2.5_ and PM_2.5_ components from 2003 through 2007 are shown in Table 1. Average daily concentrations for PM_2.5_, EC, OC, sulfate, and nitrate were 7.98, 0.47, 3.09, 1.08, and 1.03 μg/m^3^, respectively. Nitrate and EC showed the most clear seasonal cyclical pattern with both having higher winter concentrations (Fig. 2). Nitrate concentrations were particularly low in summer. Table 2 shows crude correlations of all pollutants and meteorological measures. Daily concentrations of PM_2.5_ were strongly correlated with those of nitrate, somewhat less strongly correlated with sulfate and OC, and moderately correlated with EC. EC and OC were relatively strongly correlated as were sulfate and nitrate. There was the high correlation of PM_2.5_ with nitrate, but distinctively low correlation with NO_2_, along with the negative correlation with temperature. Mortality data for the same time period are shown in Table 1. Average daily total mortality was 33 deaths/day (standard deviation = 6.2), with 11 (3.4), 2 (1.3), 4 (2.1), and 8 (2.9) deaths/day attributed to cardiovascular, IHD, respiratory, and cancer causes, respectively. Time-series plots of mortality show that death counts based on cardiovascular and respiratory causes were higher in the winter than the summer (Fig. 2).

**Table 1:** Descriptive data on daily PM_2.5_ mass and four PM_2.5_ component concentrations, gaseous pollutants, meteorology and daily mortality counts in the 5-county Denver metropolitan region from 2003 through 2007

		N	Mean	SD^*^	Minimum	Median	Maximum	IQR^*^
Pollutant	PM_2.5_	1808	7.98	5.08	−0.92	6.87	59.41	4.54
(μg/m^3^)^+^	EC^*^	1809	0.47	0.33	0.00	0.40	3.02	0.33
	OC^*^	1809	3.09	1.39	−0.78	2.91	10.28	1.67
	Sulfate	1808	1.08	0.97	0.00	0.91	14.32	0.76
	Nitrate	1808	1.03	1.97	−0.02	0.22	19.72	0.86
Gaseous	CO^*^ (ppm)	1779	1.47	0.97	0.40	1.20	15.00	0.80
pollutant^+^	SO_2_^*^ (ppb)	1675	10.53	7.66	0.00	9.00	60.00	9.00
	NO_2_^*^ (ppb)	1578	46.91	14.33	9.00	46.00	136.00	18.00
	Ozone (ppb)	1729	39.70	16.97	0.00	40.25	108.63	24.63
Weather^+^	Temperature (deg F)	1813	51.09	17.43	−4.60	51.50	85.40	27.75
	Relative humidity (%)	1813	54.62	20.77	14.25	51.83	100.00	30.46
Mortality	Total	1826	33.2	6.2	14	33	54	8
(N)	Cardiovascular	1826	11.0	3.4	2	11	27	4
	Ischemic heart disease	1826	1.6	1.3	0	1	8	1
	Respiratory	1826	3.9	2.1	0	4	14	3
	Cancer	1826	8.4	2.9	1	8	20	4

*SD = standard deviation; IQR = inter-quartile range ; EC = elemental carbon; OC = organic carbon; CO = carbon monoxide; NO_2_; nitrogen dioxide; SO_2_ = sulfur dioxide

^+^PM_2.5_, four PM_2.5_ components, and meteorology were measured at the DASH site, whereas gaseous pollutants were collected at two EPA sites in Fig. 1

**Table 2:** Pearson correlation coefficients of PM_2.5_, four PM_2.5_ components, gaseous pollutants, and meteorological measures in the 5-county Denver metropolitan region from 2003 through 2007

	EC^*+^	OC^*+^	Sulfate^+^	Nitrate^+^	CO^*+^	SO_2_^*+^	NO_2_^*+^	Ozone^+^	Temperature^+^	Humidity^+^
PM_2.5_^+^	0.46	0.54	0.68	0.82	0.23	0.23	0.26	−0.30	−0.23	0.34
EC		0.55	0.09	0.36	0.63	0.35	0.37	−0.47	−0.20	0.02
OC			0.20	0.26	0.30	0.31	0.32	0.00	0.18	−0.14
Sulfate				0.56	−0.03	0.01	−0.01	−0.06	−0.05	0.37
Nitrate					0.20	0.14	0.16	−0.49	−0.53	0.47
CO						0.34	0.50	−0.46	−0.23	−0.08
SO_2_							0.34	−0.13	0.06	−0.20
NO_2_								−0.14	−0.08	−0.23
Ozone									0.70	−0.39
Temperature										−0.52

* EC = elemental carbon; OC = organic carbon; CO = carbon monoxide; NO_2_; nitrogen dioxide; SO_2_ = sulfur dioxide

+ PM_2.5_, four PM_2.5_ components, and meteorology were measured at the DASH site, whereas gaseous pollutants were collected at two EPA sites in Fig. 1

**Fig. 3:**
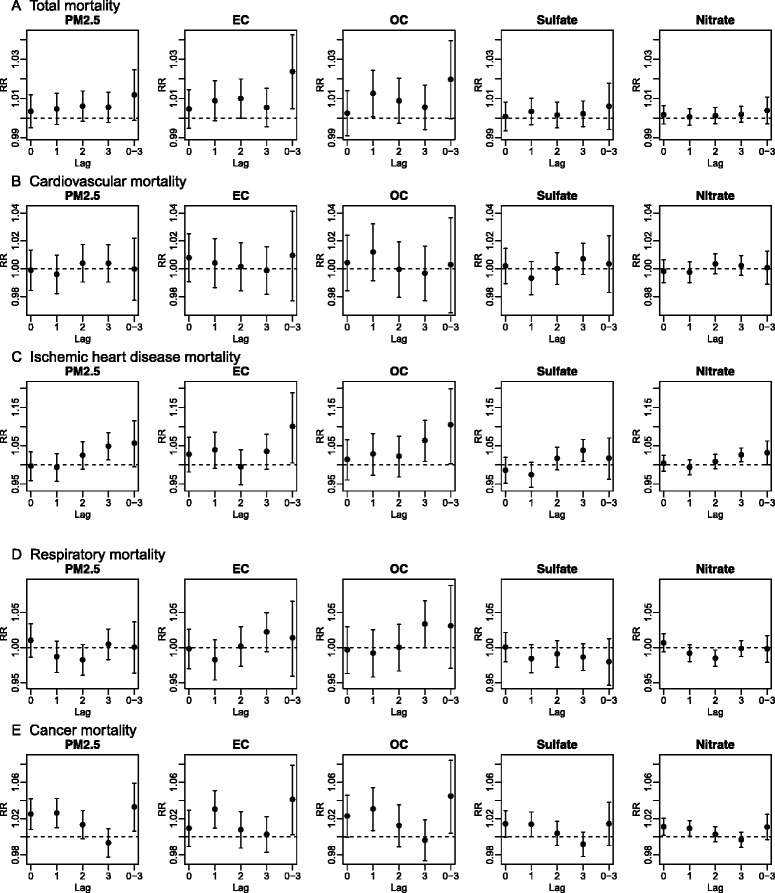
Relative risks and 95 % confidence intervals of (A) total, (B) cardiovascular, (C) ischemic heart disease, (D) respiratory, and (E) cancer mortality for inter-quartile increases in PM_2.5_ and four PM_2.5_ components (4.54, 0.33, 1.67, 0.76, and 0.86 μg/m^3^ for PM_2.5_, EC, OC, sulfate, and nitrate, respectively) by lag in the 5-county Denver metropolitan region from 2003 through 2007

Estimated RRs of non-accidental total, cardiovascular, IHD, respiratory, and cancer mortality for IQR increases in PM_2.5_ and each of the PM_2.5_ component concentrations are shown in Table 3 and Fig. 3. IQRs of PM_2.5_, EC, OC, sulfate, and nitrate concentrations were 4.54, 0.33, 1.67, 0.76, and 0.86 μg/m^3^, respectively. Total mortality was associated with PM_2.5_, EC, and OC (RR = 1.01 [95 % CI = 1.00, 1.01], 1.02 [1.01, 1.04], and 1.02 [1.01, 1.04], respectively) at lag 0–3. The estimated RRs of EC and OC were generally larger, particularly at lags 1, 2 and 0–3 than those of the other PM_2.5_ components and of PM_2.5_. There was little evidence of associations of PM_2.5_ or PM_2.5_ components on cardiovascular and respiratory mortality. However, PM_2.5_ and all four PM_2.5_ components were associated with IHD mortality at lag 3 or lags distributed over days 0–3. RRs for IHD were higher than those of any other cause-specific mortality, but with much wider CIs. Estimated RRs of cancer mortality were higher than those of cardiovascular or respiratory mortality. PM_2.5_ as well as all four PM_2.5_ components were associated with cancer mortality at lags 0, 1, or 0–3 with larger effect estimates for EC and OC.

**Table 3:** Relative risks (RRs) and 95 % confidence intervals (CIs) of total, cardiovascular, ischemic heart disease, respiratory, and cancer mortality for an inter-quartile increases in PM_2.5_ and four PM_2.5_ components (4.54, 0.33, 1.67, 0.76, and 0.86 μg/m^3^ for PM_2.5_, EC, OC, sulfate, and nitrate, respectively) at lag 0–3 from unconstrained distributed lag models in the 5-county Denver metropolitan region from 2003 through 2007

	Mortality														
	Total			Cardiovascular			IHD^*^			Respiratory			Cancer		
Pollutant	RR	95 % CI		RR	95 % CI		RR	95 % CI		RR	95 % CI		RR	95 % CI	
PM_2.5_	1.012	(0.999,	1.025)	1.000	(0.978,	1.022)	1.057	(0.998,	1.119)	1.010	(0.974,	1.048)	1.033	(1.007,	1.060)
EC^*^	1.024	(1.005,	1.043)	1.010	(0.978,	1.042)	1.100	(1.013,	1.196)	1.026	(0.973,	1.081)	1.041	(1.004,	1.080)
OC^*^	1.020	(1.000,	1.040)	1.003	(0.969,	1.038)	1.105	(1.011,	1.207)	1.038	(0.980,	1.099)	1.045	(1.005,	1.086)
Sulfate	1.006	(0.994,	1.018)	1.004	(0.983,	1.024)	1.018	(0.965,	1.073)	0.992	(0.959,	1.027)	1.014	(0.991,	1.038)
Nitrate	1.004	(0.997,	1.011)	1.001	(0.989,	1.013)	1.032	(1.001,	1.063)	1.002	(0.983,	1.021)	1.011	(0.997,	1.025)

* EC = elemental carbon; OC = organic carbon; IHD = ischemic heart disease

**Fig. 4:**
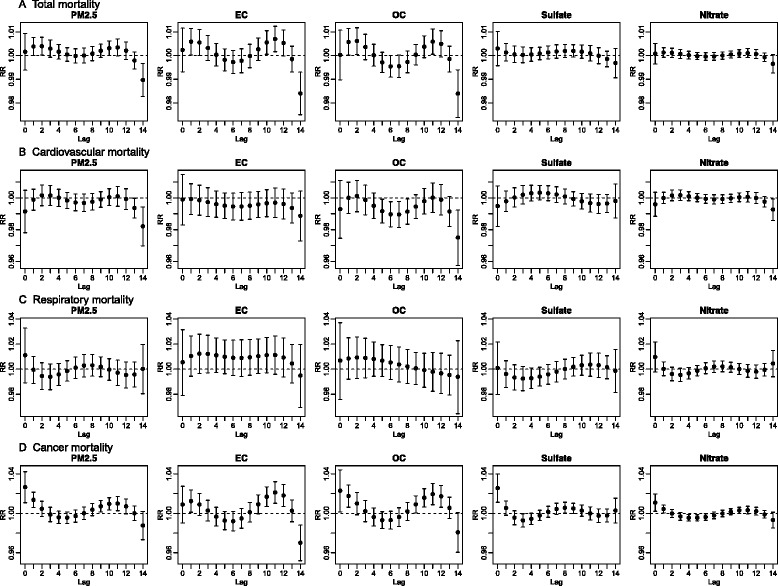
Temporal patterns of relative risks of (A) total, (B) cardiovascular, (C) respiratory, and (D) cancer mortality for inter-quartile increases in PM_2.5_ and four PM_2.5_ components (4.54, 0.33, 1.67, 0.76, and 0.86 μg/m^3^ for PM_2.5_, EC, OC, sulfate, and nitrate, respectively) from lag 0 through lag 14 from the constrained distributed lag models in the 5-county Denver metropolitan area from 2003 through 2007

In sensitivity analyses, there were no meaningful changes in effect estimates when meteorology and temperature were modeled differently (see Methods), or the number of df for the time smooth was increased or decreased (data not shown). Results were also robust to adjustment for barometric pressure, PM_2.5_ residuals and gaseous pollutants, and restriction of the study area to 10 and 20 kilometer buffers (Additional file 1: Figure S1). The two pollutant models adding another PM_2.5_ component gave largely consistent results although with some sensitivity of effect estimates for EC and OC, and for sulfate and nitrate, when adjusted for effects of the other. Findings for total and cause-specific mortality were generally consistent in strata of age and sex. Effect estimates varied by season but did not show any consistent patterns with wide CIs particularly in the summer (Additional file 1: Figure S2). In the investigation of lag structure, estimated RRs for cancer mortality over lag 0 through 14 were positive at lags 0 and 1, became negative between lags 2 and 7 and increased afterwards (Fig. 4), suggesting mortality displacement [18, 19].

## Discussion

The DASH study features daily PM_2.5_ speciation data measured over the five years that allow full use of daily mortality data, as well as the ability to estimate the total effect of a pollutant component over a range of days (the distributed lag) rather than an effect of one day in isolation. Findings show suggestive associations with total mortality, especially for EC and OC, but little evidence of associations specifically with cardiovascular or respiratory mortality; the clearest associations were seen for cancer mortality particularly for EC and OC with possibly mortality displacement. PM_2.5_, EC and OC associations distributed over several days were somewhat larger than those at any single day lag.

We found no evidence for associations between cardiovascular mortality and PM_2.5_ or the PM_2.5_ components, whereas total mortality was associated with EC and OC. For PM_2.5_, no associations of cardiovascular mortality were commonly found in single-city time-series studies performed in the U.S [14]. A meta-analysis study across 27 U.S. communities also reported a short-term association with total mortality but no association with cardiovascular mortality [20]. Another meta-analysis study in 57 U.S. cities, however, found a short-term association with cardiovascular mortality but stronger association with total mortality [21]. As opposed to single-city time-series studies of PM_2.5_, a few recent time-series studies focusing on PM_2.5_ components found higher effect estimates for cardiovascular mortality than total mortality. Ostro et al. (2007) found generally higher effect estimates of PM_2.5_, EC, OC and nitrate in California on cardiovascular mortality than total mortality at lag 0 through 3 [1]. In Seattle, stronger associations were seen for cardiovascular mortality with PM_2.5_ and EC than for total mortality in the cold season [4]. While these two studies had generally larger numbers of daily deaths for cardiovascular diseases than ours, suggesting that power may have been better, the daily sampling of PM_2.5_ components for 5 years actually resulted in comparable or better power in the DASH study compared to those studies which used regulatory monitoring data measured every 3rd or 6th day for 4 and 3 years, respectively. It is possible that there is still insufficient power in single city studies, however, in light of the evidence of associations for PM_2.5_ from multi-city studies [21]. We did see associations with IHD mortality with larger RRs than those of other causes of death. However, the effect estimates for IHD mortality had wider CIs and were based on sparse outcome data (median of 1 death/day), so the size of the IHD RRs should be interpreted cautiously. In order to determine whether cardiovascular mortality associations might be observed using immediate cause of death codes rather than underlying cause of death codes, we repeated the analyses for immediate causes of death. Again, no associations with cardiovascular death were seen (data not shown). Future studies expanded to other areas and multi-city settings may allow for better assessment of the association of PM_2.5_ component with cause-specific mortality.

The associations with all cancer mortality were stronger for PM_2.5_ and the four PM_2.5_ components than for any other cause-specific mortality, with the exception of IHD mortality, as noted above. Higher effect estimates of total mortality than those of cardiovascular and respiratory mortality may indicate stronger associations with other common causes of mortality, such as cancer mortality. Higher effect estimates of cancer mortality than of non-cancer mortality (Additional file 1: Figure S3), as suggested by the findings in Fig. 3, also indicate that cancer mortality contributes to the effect estimates for total mortality. Ours is not the only study reporting stronger associations with cancer mortality than other cause-specific mortality. Studies performed in Europe and Canada have reported similar findings for PM_2.5_ and coefficient of haze [22, 23]. Obviously short term exposure to air pollution does not cause cancer in the immediately following days. Generally high effect estimates in the four most common cancer types compared to those of cardiovascular and respiratory mortality suggest that the associations with cancer mortality was not due to a specific type of cancer, and may indicate an effect on something common to most cancers. Postulated mechanisms for acute effects of particulate air pollution such as autonomic modulation or pulmonary inflammation causing systemic inflammation [24] could conceivably place additional stress on the extremely frail members of the population, such as those at imminent risk of dying from an underlying malignancy, causing advancement in the time of death. We found larger effect estimates of cancer mortality at lags 0 and 1, which then decreased quickly and became negative at 2 to 7 lag days, depending on the component (Fig. 4). This finding suggests a possibility of increased deaths in a high risk pool of a susceptible population immediately following daily increases in PM_2.5_ with depletion of the high risk pool resulting in subsequent negative effect estimates, a phenomenon known as “mortality displacement” or “harvesting” [18, 19]. Although this frail population is likely to stay indoors more than other population, they nevertheless experience exposure to ambient PM_2.5_, a large fraction of which penetrates into indoor environments [25].

We found stronger associations for EC and OC than the inorganic secondary ions, sulfate and nitrate. Our findings on EC and OC are consistent with previous time-series studies focusing on PM_2.5_ components performed in New York City, Phoenix, California, and Seattle which also showed stronger associations for EC and/or OC than other PM_2.5_ components [1–4]. Other epidemiological and toxicological studies also supported the associations of EC and OC [26, 27]. EC and OC have been considered markers for combustion sources such as traffic. In particular, EC has been of particular interest as a marker of diesel exhaust, although other combustion sources such as wood burning also contribute to EC [28, 29]. In Denver, there are strong indications that EC reflects primarily diesel emissions [30]. Most convincingly, daily EC concentrations were lower on weekends in Denver than during the week [31], a pattern that parallels the intensity of diesel traffic throughout the week. Although OC represents traffic emission, a large proportion of OC consists of secondary organic aerosols as well. A previous DASH source apportionment showed that the addition of organic molecular markers and estimated semi-volatile organic compounds improved source characterization for identifying primary vs. secondary and vehicle vs. non-vehicle combustion sources [32, 33]. We plan to adopt these source apportionment outputs in heath analysis in order to better understand health effects of specific sources. The current findings suggest hypotheses that could be addressed with the addition of this much richer PM component data. As opposed to Ostro et al. (2011), we found little evidence of associations for nitrate. These results could be in part due to poor measurements related to volatilization in summer with high temperature and low humidity in the Denver area. Thirty eight percent of the nitrate measurements were below the limit of detection [13]. Different population characteristics and statistical models could also have affected these findings. However, our sensitivity analyses including stratified analyses by sex and age groups and alternative statistical models were reassuring in this regard.

This study has some limitations and implications for future studies. Because air pollution concentrations were measured at only one site in the Denver metropolitan region, it is likely that these population exposures to ambient air pollution were estimated with some degree of error. Measurement error might be expected to be greater for those PM_2.5_ components such as EC and OC that are more affected by local sources than are total PM_2.5_ mass or secondary sulfate and nitrate components. However, in a supplemental study in which sampling was carried out in 2009 at three additional sites in the Denver area in addition to the site described in this study, we found little spatial heterogeneity of the PM_2.5_ components time series or for pollution sources estimated by positive matrix factorization (PMF) across the four sites [34, 35]. We were also reassured by preliminary analyses of the DASH site showing that our PM_2.5_ component concentrations were highly correlated (0.70-0.86) over time with those at an EPA Chemical Speciation Network site located 13 kilometers away (Fig. 1). Some previous studies found associations of mortality with trace metals such as copper and zinc [1, 3]. However, we did not include metal components in our health analysis because only one year of sampling for trace metals in DASH would limit the power to detect associations.

Here we report mortality findings relating to the bulk PM_2.5_ species. A detailed set of speciated organic compounds was also measured daily at the DASH monitoring site [32, 36]. In future analyses we intend to incorporate PMF source estimates of exposure making use of both the bulk species and the speciated organic compounds [32, 33].

## Conclusions

Short-term exposure to increased concentrations of some PM_2.5_ components was associated with increased daily mortality. Associations were stronger for EC and OC than for sulfate and nitrate, secondary inorganic aerosols, especially in those with cancer as an underlying cause of death. Individuals with cancer may represent a very frail subpopulation whose deaths could be advanced given high exposures to PM_2.5_ component air pollution. Future studies are needed to identify the specific sources of air pollution, such as traffic emissions, for example, that are more responsible for producing these adverse effects.

## Additional file

Additional file 1: Figures S1, S2, and S3. Supplemental Figures of the ‘Components of fine particulate matter and daily mortality in the Denver Aerosol Sources and Health (DASH) study’.

## Abbreviations

DASH: Denver Aerosol Sources and Health
DF: Degrees of Freedom
EC: Elemental Carbon
EPA: Environmental Protection Agency
ICD-10: International Classification of Diseases 10th Revision
IHD: Ischemic Heart Disease
IQR: Interquartile Range
OC: Organic Carbon
PMF: Positive Matrix Factorization
PM_2.5_: Particulate Matter with aerodynamic diameter less than or equal to 2.5 μm
RR: Relative Risk
95 % CI: 95 % Confidence Interval
