# Erratum to: The short-term association of selected components of fine particulate matter and mortality in the Denver Aerosol Sources and Health (DASH) study

**DOI:** 10.1186/s12940-016-0169-1

**Published:** 2016-08-09

**Authors:** Sun-Young Kim, Steven J. Dutton, Lianne Sheppard, Michael P. Hannigan, Shelly L. Miller, Jana B. Milford, Jennifer L. Peel, Sverre Vedal

**Affiliations:** 1 Department of Environmental and Occupational Health Sciences, University of Washington School of Public Health, Seattle, WA USA; 2 Institute of Healthand Environment, Seoul National University, Seoul, Korea; 3 National Center for Environmental Assessment, U.S. Environmental Protection Agency, RTP, NC USA; 4 Department of Biostatistics, University of Washington School of Public Health, Seattle, WA USA; 5 Departments of Mechanical Engineering, College of Engineering and Applied Science, University of Colorado, Boulder, CO USA; 6 Department of Environmental and Radiological Health Sciences, Colorado State University, Fort Collins, CO USA

## Erratum

Unfortunately, in the original version of this article [1] the Acknowledgements section included wrong funding information and gave the grant number for “the National Research Foundation of Korea” as “900-20140076” instead of “2013R1A6A3A04059017”. The corrected Acknowledgements have been included in full in this erratum.

## Acknowledgements

This work was supported by the National Institute of Environmental Health Sciences (R01 ES010197), the U.S. Environmental Protection Agency (RD 834796), and the National Research Foundation of Korea (Basic Science Research Program through the National Research Foundation of Korea funded by the Ministry of Education: 2013R1A6A3A04059017).
