# Supplementary material for: The short-term association of selected components of fine particulate matter and mortality in the Denver Aerosol Sources and Health (DASH) study
Source: Environ Health. 2015 Jun 6;14:49. doi: 10.1186/s12940-015-0037-4 (PMC4456999; doi:10.1186/s12940-015-0037-4)
Supplement: Additional file 1: Figures S1, S2, and S3. — Supplemental Figures of the ‘Components of fine particulate matter and daily mortality in the Denver Aerosol Sources and Health (DASH) study’. [file 12940_2015_37_MOESM1_ESM.pdf]

Supplemental Figures of the ‘Components of fine particulate matter and daily mortality in the Denver Aerosol Sources and Health (DASH) study’

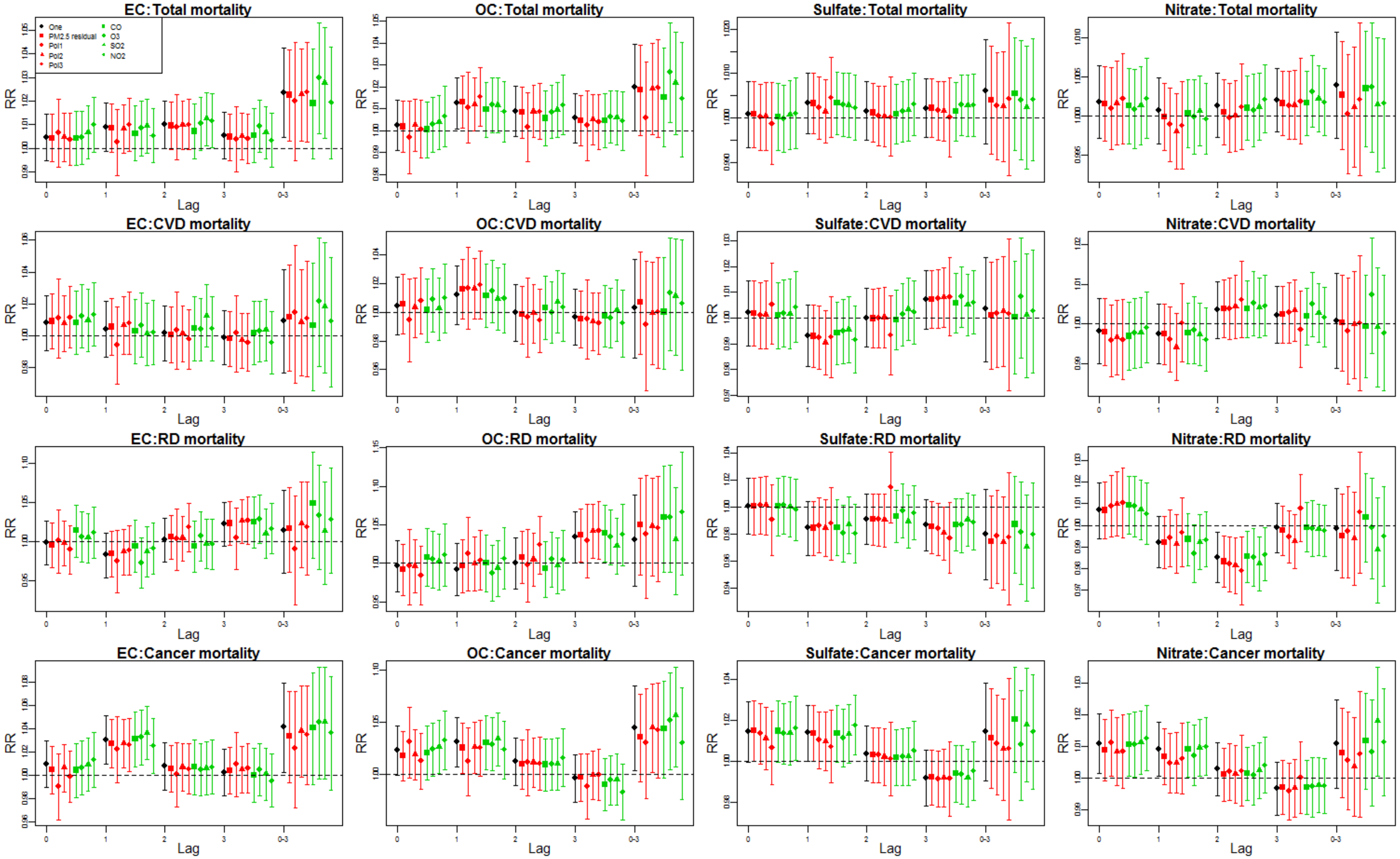

Supplemental Figure 1. Relative risks and 95% confidence intervals of total, cardiovascular disease (CVD), respiratory disease (RD), and cancer mortality for inter-quartile increases in four $PM_{2.5}$ components (0.33, 1.67, 0.76, and 0.86 $\mu g/m^3$ for EC, OC, sulfate, and

nitrate, respectively) adjusting for $PM_{2.5}$ residuals, another $PM_{2.5}$ components or gaseous pollutants in two pollutant models (one: original one pollutant model; Pol1 to Pol3: three pollutants out of the four in the order of EC, OC, sulfate and nitrate) at lags 0, 1, 2, 3, and 0-3 in the 5-county Denver metropolitan region from 2003 through 2007.

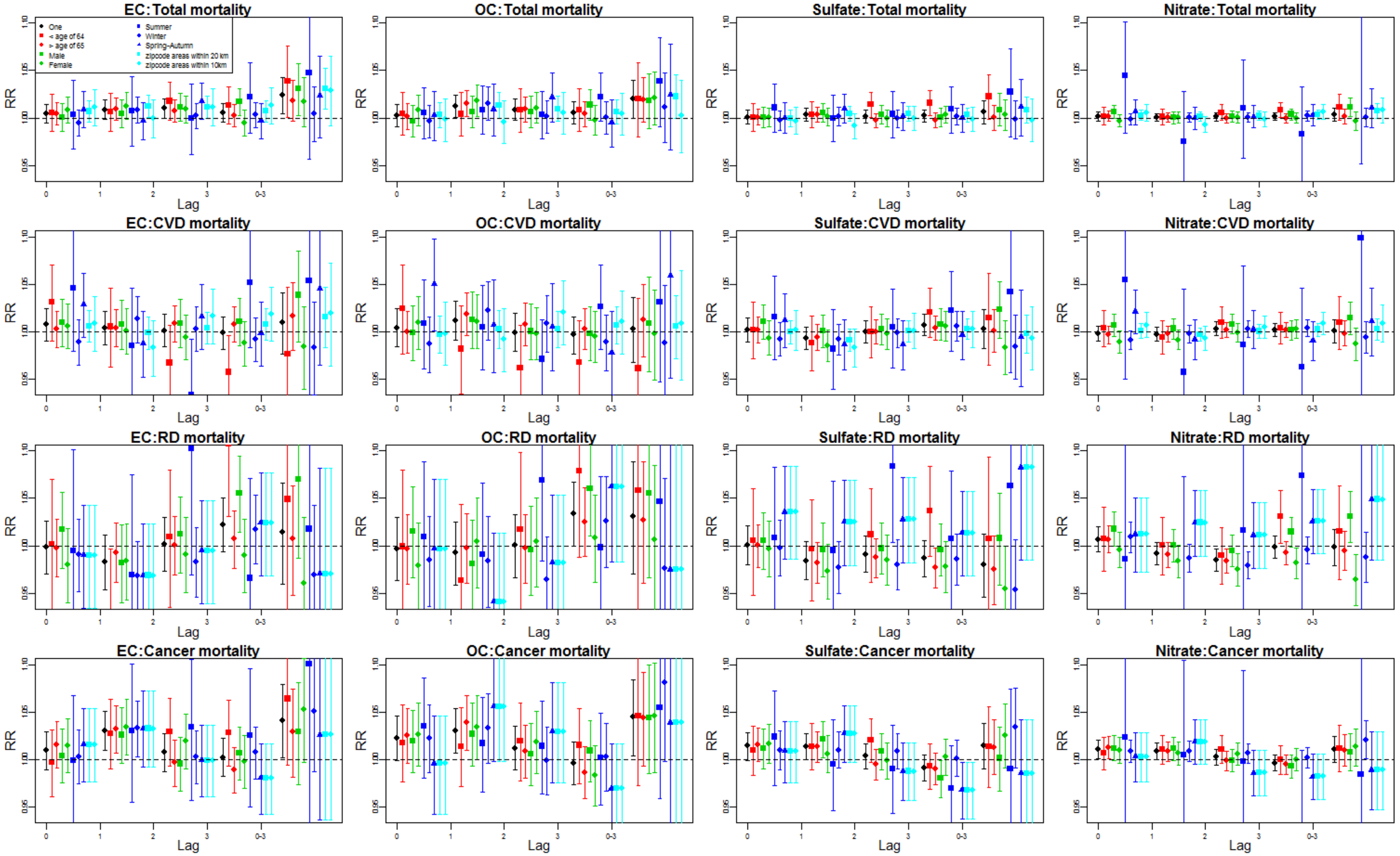

Supplemental Figure 2. Relative risks and 95% confidence intervals of total, cardiovascular disease (CVD), respiratory disease (RD),

and cancer mortality for inter-quartile increases in four $PM_{2.5}$ components (0.33, 1.67, 0.76, and 0.86 μg/m^3 for EC, OC, sulfate, and nitrate, respectively) stratified by age, sex, season, and area at lags 0, 1, 2, 3, and 0-3 in the 5-county Denver metropolitan region from 2003 through 2007.

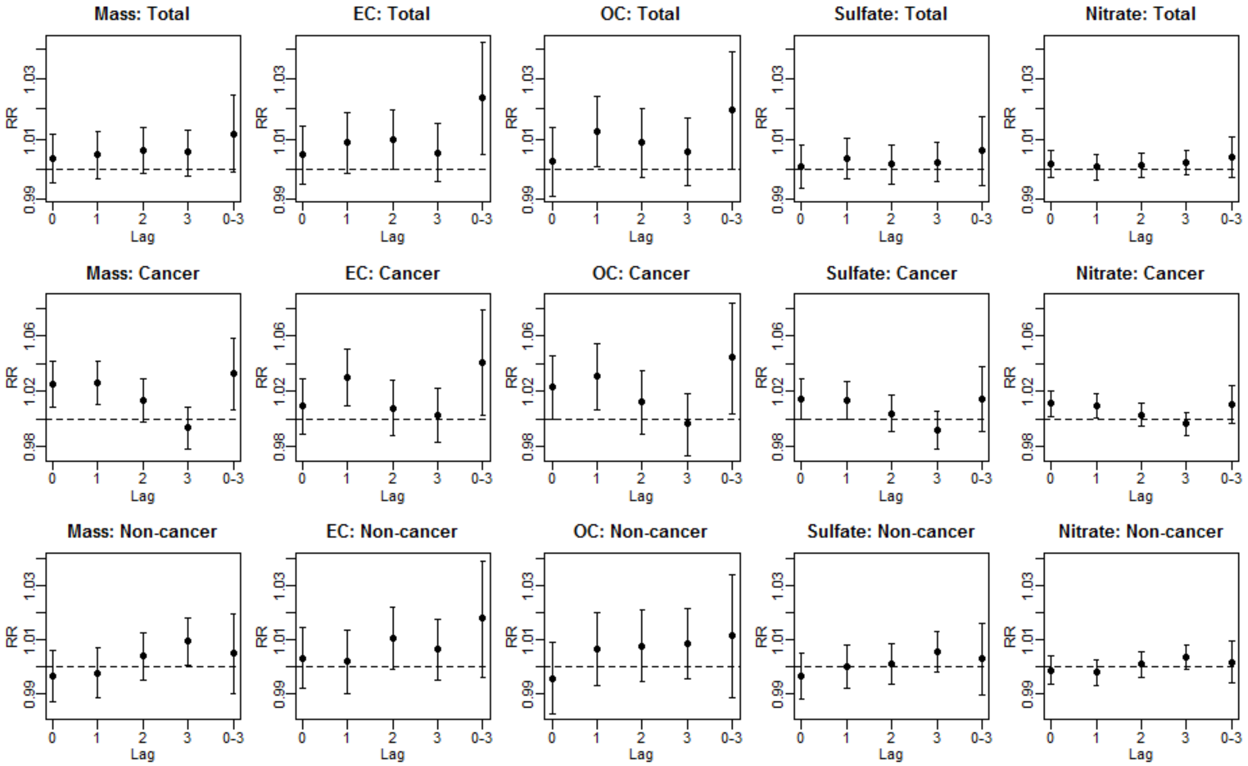

Supplemental Figure 3. Relative risks and 95% confidence intervals of total, cancer, and non-cancer mortality for inter-quartile increases in $PM_{2.5}$ and four $PM_{2.5}$ components (4.54, 0.33, 1.67, 0.76, and 0.86 μg/m^3 for EC, OC, sulfate, and nitrate, respectively)
